# Continuing Medical Education Information

**DOI:** 10.1093/noajnl/vdaa072

**Published:** 2020-06-25

## Neurofibromatosis in *Neuro-Oncology Advances*

**Release Date:** June 25, 2020

**Last Reviewed:** May 20, 2020

**Expiration Date:** June 25, 2021

**Time to Complete Activity:** 4.0 Hours

## Instructions for Participation

To receive a CME certificate of participation, you should:

- Read the entire publication, including the CME information
- Register or log in at www.paradigmmc.com/a942 to complete and submit the online posttest and evaluation

Following online completion of the posttest and evaluation, a certificate of participation will be available for download/printing immediately. For questions regarding CME credit, contact the Paradigm CME Department at (845) 398–5949.

There is no fee required for participation in this activity.

This activity is provided by Paradigm Medical Communications, LLC.
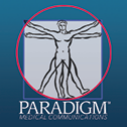


## Online Requirements to View Activity

### Browser Compatibility

The Oxford Academic website is optimized for use with currently supported, secure versions of these browsers: Chrome, Firefox, Internet Explorer, and Safari. It can be accessed using older versions, but some features may not function properly. Please check your browser or contact your organization’s IT department for details of any browser updates available.

The Oxford Academic website uses third-party plug-in software to display certain types of content. The third-party software may have features that are not supported by all browsers. Where possible, Oxford University Press will work with the third party to address browser-specific issues.

### Adobe Reader

This activity is in Adobe Acrobat PDF format. To view the activity, you will need Adobe Reader. If you do not have Adobe Reader installed on your computer, please visit https://get.adobe.com/reader/ to install a free version of Adobe Reader.

### Technical Support

If you have any technical problems, email us at contactus@paradigmmc.com.

### Target Audience

This activity has been designed to address the educational needs of neuro-oncologists. It may also be of benefit to other clinicians interested or involved in the treatment of patients with neurofibromatosis.

### Learning Objectives

Upon proper completion of this activity, participants should be better able to:

- Describe the relevance of the RAS/MAPK signaling pathway in the pathogenesis of NF1 and the mechanism of action of MEK inhibitors
- Interpret efficacy and safety data from the latest clinical trials investigating MEK inhibitors for treatment of neurofibromas
- Describe the molecular drivers for the spectrum of tumors in NF1
- Assess the development of potential targeted therapies for the spectrum of tumors in NF1
- Describe the importance of developing new measures of quality of life to facilitate the design of clinical trials in NF1

### Physician Accreditation Statement

Paradigm Medical Communications, LLC is accredited by the Accreditation Council for Continuing Medical Education to provide continuing medical education for physicians.

### Physician Credit Designation Statement

Paradigm Medical Communications, LLC designates this enduring material for a maximum of 4.0 *AMA PRA Category 1 Credits*™. Physicians should claim only the credit commensurate with the extent of their participation in the activity.

### Disclosure of Commercial Support

This activity is supported by an educational grant from AstraZeneca.

### Guest Editor

**Suganth Suppiah, MD**

Resident Physician

Department of Neurosurgery

University of Toronto

Toronto, ON, Canada

**Table:** 

Authors		
**Tyler Ball** Inpatient Pharmacy Technician Columbia, MO	**Carolina Barnett-Tapia, MD, PhD** Assistant Professor of Medicine, Neurology University of Toronto Prosserman Centre for Neuromuscular Diseases Toronto General Hospital Toronto, ON, Canada	**Jeffrey Berman, PhD** Research Assistant Professor Radiology Children’s Hospital of Philadelphia Philadelphia, PA
**Gabriela Bernal** Department of Pathology Vall d’Hebron University Hospital Barcelona, Spain	**Josep Biayna, PhD** Postdoctoral Researcher Institute for Research in Biomedicine of Barcelona Barcelona, Spain	**Jaishri Blakely, MD** Marjorie Bloomberg Tiven Professor of Neurfibromatotis Johns Hopkins University School of Medicine Baltimore, MD
**Ignacio Blanco, MD** Clinical Director, Laboratory Clinic Metropolitana Nord Chairman, Clinical Genetics Department Hospital Germans Trias Baldalona, Barcelona, Spain	**Audrey Briand-Suleau, PhD** Team Genomics and Epigenetics of Rare Tumors Institut Cochim Université de Paris Paris, France	**Vera Bril, MD** Professor Elisabeth Raab Neurofibromatosis Clinic University Health Network University of Toronto Toronto, ON, Canada
**Laurence Brugiès, MD** Department of Oncology for Children and Adolescents Gustave Roussy Cancer Center Paris-Saclay University Villejuif, France	**Steven L. Carroll, MD, PhD, FASCP, FCAP** Professor and Chair Gordon R. Hennigar Jr, MD Endowed Chair in Pathology Chief, Pathology and Lab Medicine ICCE Director, Carroll A. Campbell Jr Brain Bank Director, Hollings Cancer Center Biorepository and Tissue Analysis Shared Resources Medical University of South Carolina Charleston, SC	**Bahir H. Chamseddin, MD** UT Southwestern Medical School Dallas, TX
**Zhiguo Chen, MD, PhD** Instructor Department of Dermatology UT Southwestern Medical Center Dallas, TX	**Xin Chou, PhD** Shanghai Medical College Fudam University Shanghai, PR, China	**John Chrisinger, MD** Assistant Professor Section Head, Bone and Soft Tissue Pathology Department of Pathology and Immunology Washington University School of Medicine St. Louis, MO
**Sonika Dahiya, MD** Associate Professor Division of Neuropathology Department of Pathology and Immunology Washington University School of Medicine St. Louis, MO	**Anna J. Dare, MBChB, PhD** General Surgery Resident Department of Surgery University of Toronto Toronto, ON, Canada	**Amanda de Andreade Costal, MSc** Neurofibromatosis Center Washington University School of Medicine St. Louis, MO
**Peter de Blank, MD, MSCE** Associate Professor University of Cincinnati and Cincinnati Children’s Hospital Medical Center Cincinnati, OH	**Hilary K. Dietz, MD** Resident Physician Department of Internal Medicine Barnes Jewish Hospital Washington University School of Medicine St. Louis, MO	**Li Ding, PhD** Professor of Medicine Washington University School of Medicine St. Louis, MO
**Marco A. Fernández, BSc** Head of Cytometry Core Facility Germans Trias and Pujol Research Institute Barcelona, Spain	**Juana Fernández-Rodríquez, PhD** Hereditary Cancer Program Catalan Institute of Oncology Barcelona, Spain	**Marc Ferrer, PhD** National Center for Advancing Translational Sciences National Institute for Health Chemical Genomics Center Bethesda, MD
**Michael J. Fisher, MD** Chief, Neuro-Oncology Section Director, Neurofibromatosis Program Hubert J.P. and Anne Faulkner Schoemaker Endowed Chair in Pediatric Neuro-Oncology Professor of Pediatrics Center for Childhood Cancer Research and Division of Oncology The Children’s Hospital of Philadelphia Philadelphia, PA	**Jonathan S. Fletcher** MD/PhD student Medical Scientist Training Program University of Cincinnati College of Medicine Cincinnati, OH	**Yashan Gao, MD** Department of Plastics and Reconstructive Surgery Shanghai Ninth People’s Hospital Shanghai Jiao Tong University School of Medicine Shanghai, PR, China
**Bernat Gel, PhD** Bioinformatician Hereditary Cancer Group Germans Trias i Pujol Institute Badalona, Barcelona, Spain	**Rebecca A. Gladdy, MD, PhD, FRCSC, FACS** Clinician-Scientist Lunenfeld-Tanenbaum Research Institute Associate Professor of Surgery Division of Surgical Oncology Mount Sinai Hospital Princess Margaret Care Centre University of Toronto Toronto, ON, Canada	**Jacques Grill, MD, PhD** Head of Pediatric Tumor Board Team Leader, INSERM U981 Genomics and Oncogenesis of Brain Tumor Gustave Roussy Villejuif, France
**Abigail Godec** MD/ PhD Student Michigan State University College of Human Medicine Van Andel Graduate School East Lansing, MI	**Andrea M. Gross, MD** Assistant Research Physician Pediatric Oncology Branch National Cancer Institute Bethesda, MD	**Bin Gu, MD** Chief Surgeon Department of Plastic and Reconstructive Surgery Shanghai Ninth People’s Hospital Shanghai Jiao Tong University School of Medicine Shanghai, PR, China
**Shuchen Gu, MD** Department of Plastic and Reconstructive Surgery Shanghai Ninth People’s Hospital Shanghai Jiao Tong University School of Medicine Shanghai, PR, China	**Léa Guerrini-Rousseau, MD** Department of Pediatric and Adolescent Oncology Gustave Roussy Cancer Center Paris-Saclay University Villejuif, France	**Rajarshi Guha, PhD** National Center for Advancing Translational Sciences National Institute of Health Chemical Genomics Centre Bethesda, MD
**Abha Gupta, MD, MSc, FRCPC** Associate Professor, Department of Pediatrics University of Toronto Staff Oncologist, Solid Tumour Program The Hospital for Sick Children Staff Oncologist, Sarcoma Program Princess Margaret Cancer Centre Medical Director, Adolescent and Young Adult Program Toronto, ON, Canada	**David H. Gutmann, MD, PhD, FAAN** Donald O. Schnuck Family Professor Vice Chair for Research Affairs Department of Neurology Director, Neurofibromatosis Center Washington University School of Medicine St. Louis, MO	**Geohana Hamoy-Jimenez, MD** Division of Neurology University Health Network University of Toronto Toronto, ON, Canada
**Angela C. Hirbe, MD, PhD** Assistant Professor of Medicine Washington University School of Medicine Sarcoma Program Neurofibromatosis Program St. Louis, MO	**Xin Huang, MD** Department of Plastic and Reconstructive Surgery Shanghai Ninth People’s Hospital Shanghai Jio Tong University School of Medicine Shanghai, PR, China	**Reyka Jayasinghe, PhD** Postdoctoral Research Associate Department of Medicine Washington University School of Medicine St. Louis, MO
**Ren JieYi, MD** Department of Plastic and Reconstructive Surgery Shanghai Ninth People’s Hospital Shanghai Jio Tong University School of Medicine Shangahi, PR, China	**Madhurima Kaushal, MS** Bioinformaticist Institute for Informatics Washington University School of Medicine St. Louis, MO	**Raymond H. Kim, MD, PhD, FRCPC, FCCMG, FACMG** Medical Geneticist University Health Network Toronto, ON, Canada
**Adam Lane, PhD** Assistant Professor, Department of Pediatrics University of Cincinnati Cincinnati, OH	**Ingrid Laurendeau** Engineer Paris Descartes University Paris, France	**Conxi Lázaro, PhD** Head, Molecular Diagnostic Unit Hereditary Cancer Program Institut Català d’Oncologia Barcelona, Spain
**Lu Q. Le, MD, PhD** Professor of Dermatology Simmons Comprehensive Cancer Center University of Texas Southwestern Medicine Center Dallas, TX	**Hai-Zhou Li** Department of Plastic and Reconstructive Surgery Shanghai Ninth People’s Hospital Shanghai Jiao Tong University School of Medicine Shanghai, PR, China	**Hua Li, PhD** Lab Manager, Wallace Lab Department of Molecular Genetics and Microbiology University of Florida Gainesville, FL
**Qing-Feng Li, MD, PhD** Department of Plastic and Reconstructive Surgery Shanghai Ninth People’s Hospital Shanghai Jiao Tong University School of Medicine Shanghai, PR, China	**Stephen Li, MD** UT Southwestern Medical Center Dallas, TX	**Xiang Lian** Researcher Shanghai Ninth People’s Hospital Shanghai Jiao Tong University School of Medicine Shanghai, PR, China
**Maria-Jeśus Lobón Iglesias, MD, PhD** Student, RTOP Team Institut Curie-Centre de Recherche Paris, France	**Jingqin Luo, PhD** Associate Professor, Siteman Cancer Center Biostatistics Shared Resource Division of Public Health Department of Surgery Washington University School of Medicine St. Louis, MO	**Zachary B. Madaj** Biostatistician II Bioinformatics and Biostatistics Core Van Andel Institute Grand Rapids, MI
**Sheila Mansouri, PhD** Scientific Associate MacFeeter-Hamilton Center for Neuro-Oncology Research Princess Margaret Cancer Center University Health Network Toronto, ON, Canada	**Helena Mazuelas Gallego** Hereditary Cancer Group Germans Trias i Pujol Research Institute Baldona, Barcelona, Spain	**Markku Miettinen, MD** Head of Surgical Pathology Laboratory of Pathology National Cancer Institute National Institute of Health Bethesda, MD
**David T. Miller, MD, PhD** Director, Multidisciplinary Neurofibromatosis Clinic Division of Genetics and Genomics Boston Children’s Hospital Boston, MA	**Douglas C. Miller, MD, PhD** Clinical Professor/Interim Chair Department of Pathology and Anatomical Sciences University of Missouri School of Medicine Columbia, MO	**Rosalynn M. Nazarian, MD** Associate Professor, Harvard Medical School Associate Pathologist, Dermatopathology Unity Pathology Service Massachusetts General Hospital Boston, MA
**Nicolas Ortonne, MD, PhD, APHP** Henri Mondor Hospital Pathology Department Paris Est Cretel University Insitut Mondor de Recherché Biomedicale Research Team Ortonne Creteil, France	**Eric Pasmant, PharmD, PhD** Department of Genetics Cochin Hospital Centre-Université de Paris and Institut Cochin Paris, France	**Melike Pekmezci, MD** Assistant Professor Departments of Pathology and Ophthalmology University of California San Francisco San Francisco, CA
**Alexander Pemov, PhD** Staff Scientist, Contractor Kelly Government Solutions Clinical Genetics Branch, Division of Cancer Epidemiology and Genetics National Cancer Institute Bethesda, MD	**Marisa Prelack, MD** Department of Neurology The Children’s Hospital of Philadelphia Philadelphia, PA	**William Presley, BSc** Lab Technician University of Florida Gainesville, FL
**Bethany C. Prudner, MD, PhD** Instructor in Medicine Division of Oncology, Department of Medicine Washington University School of Medicine St. Louis, MO	**Jay Pundavela, PhD** Research Fellow, Neural Tumors Program Experimental Hematology and Cancer Biology Cincinnati Children’s Hospital Center Early Investigator, Neurofibromatosis Program Department of Defense-Congressionally Directed Medical Research Program Cincinnati, OH	**Irma Ramos-Oliver, MD** Department of Pathology Vall d’Hebron University Barcelona, Spain
**Richa Rathore** Doctoral Candidate Washington University School of Medicine St. Louis, MO	**Nancy Ratner, PhD** Beatrice C. Lampkin Chair in Cancer Biology Program Leader, Cancer Biology and Neural Tumors Program Co-Director, Rasopathy Program Professor of Pediatrics University of Cincinnati College of Medicine Division of Experimental Hematology and Cancer Biology Cancer and Blood Diseases Institute Cincinnati Children’s Hospital Medical Center Cincinnati, OH	**Fausto Rodriguez, MD** Director, Clinical Neuropathology Service Associate Professor of Pathology and Oncology Johns Hopkins University School of Medicine Baltimore, MD
**Cleofé Romagosa, MD, PhD** Department of Pathology Vall d’Hebron University Hospital Centro de Investigación Biomédica en RED Instituto de Salud Carlos III Madrid, Spain	**Eduard Serra, PhD** Germans Trias i Pujol Research Institute Barcelona, Spain	**John R. Sollee** Clinical Research Assistant Division of Neurology Children’s Hospital of Philadelphia Philadelphia, PA
**Divya Srihari** Washington University School of Medicine St. Louis, MO	**Anat Stemmer-Rachamimov, MD** Associate Professor Department of Pathology, Division of Neuropathology Massachusetts General Hospital Harvard Medical School Boston, MA	**Yu Tao** Senior Statistical Data Analyst Washington University School of Medicine St. Louis, MO
**Arnault Tauziède-Espariat, MD** Service de Neuropathologie Sainte-Anne Paris, France	**Ernest Terribas, PhD** Program of Predictive and Personalized Medicine of Cancer Germans Trias i Pujol Research Institute Badalona, Barcelona, Spain	**Seng Thipphavong, MD, FRCPC** Abdominal Radiologist, Toronto Joint Department of Medical Imaging at University Health Network Mount Sinai Hospital Women’s College Hospital Assistant Professor, University of Toronto Toronto, ON, Canada
**Craig J. Thomas, PhD** Division of Preclinical Innovation National Center for Advancing Translational Sciences Lymphoid Malignancies Branch Center for Cancer Research National Cancer Institute National Institutes of Health Bethesda, MD	**Brian Van Tine, MD, PhD** Associate Professor of Medicine Washington University School of Medicine St. Louis, MO	**Pascale Varlet, PH** Pôle Neuro-Sainte-Ane Service Neuropathologie GHU Paris Phychiatrie & Neurosciences Paris, France
**Sharad K. Verma, PhD** Program Director Developmental Therapeutics Program National Cancer Institute Bethesda, MD	**Dominique Vidaud, MCU-PH** Hôpitaux Universitaires Paris Centre-Hôspital Cochin Service de Génétique et Biologie Moléculaires Paris, France	**Michel Vidaud, PH** Hôpitaux Universitaires Paris Centre-Hôspital Cochin Service de Génétique et Biologie Moléculaires Assistance Publique-Hôpitaux de Paris Paris, France
**Amy T. Waldman, MD, MSCE** Children’s Hospital of Philadelphia Assistant Professor of Neurology Perelman School of Medicine at the University of Pennsylvania Philadelphia, PA	**Margaret Wallace, PhD** Professor, Molecular Genetics and Microbiology UF Genetics Institute and UF Health Cancer Center University of Florida College of Medicine Gainesville, FL	**Yuxi Wang** Visiting Researcher Washington University School of Medicine St. Louis, MO
**Zhichao Wang, MD, MPH** Department of Plastic and Reconstructive Surgery Shanghai Ninth People’s Hospital Shanghai Jiao Tong University School of Medicine Shanghai, PR, China	**Cheng-jiang Wei, MD** Department of Plastic and Reconstructive Surgery Shanghai Ninth People’s Hospital Shanghai Jiao Tong University School of Medicine Shanghai, PR, China	**Brigitte Widemann, MD** Senior Investigator Head, Pharmacology and Experimental Therapeutics Section Chief, Pediatric Oncology Branch National Cancer Institute Bethesda, MD
**Xiang-Wen Xu, MD** Department of Plastics and Reconstructive Surgery Shanghai Ninth People’s Hospital Shanghai Jiao Tong University School of Medicine Shanghai, PR, China	**Gu Yihui, MD** Department of Plastics and Reconstructive Surgery Shanghai Ninth People’s Hospital Shanghai Jiao Tong University School of Medicine Shanghai, PR, China	**Gelareh Zadeh, MD** Head, Division of Surgical Oncology Department of Surgery Professor Department of Surgery University of Toronto Senior Scientist Princess Margaret Cancer Center/Ontario Cancer Institute Neurosurgeon Toronto Western Hospital Toronto, ON, Canada
**Tao Zan, MD, PhD** Shanghai Ninth People’s Hospital Shanghai Jiao Tong University School of Medicine Shanghai, PR, China	**Xiaohu Zhang** Biologist National Center for Advancing Translational Sciences National Institutes for Health Division of Preclinical Innovation Bethesda, MD	**Xiochun Zhang, MD** Washington University School of Medicine St. Louis, MO

## Disclaimer

This CME activity represents the views and opinions of the individual faculty, and does not constitute the opinion or endorsement of, or promotion by, Paradigm Medical Communications, LLC. Reasonable efforts have been taken to present educational subject matter in a balanced, unbiased fashion, and in compliance with regulatory requirements. However, each activity participant must always use his or her own personal and professional judgment when considering further application of this information, particularly as it may relate to patient diagnostic or treatment decisions including, without limitation, FDA-approved uses and any off-label uses.

As an ACCME-accredited provider, Paradigm Medical Communications, LLC’s activities must include a balanced view of therapeutic options. Use of generic names contributes to this impartiality. The use of trade names should not be viewed as an endorsement of specific products by Paradigm Medical Communications, LLC.

## Terms of Use and Privacy Policy

*Disclaimer*

The materials provided for this activity are for informational purposes only, and should not be used for diagnosis or treatment of a health problem without consulting a licensed medical professional. This CME activity represents the views and opinions of the individual faculty, and does not constitute the opinion or endorsement of, or promotion by, Paradigm Medical Communications, LLC. References to any entity, product, service, or source of information on this website should not be considered an endorsement, either direct or implied, by Paradigm Medical Communications, LLC. Paradigm Medical Communications, LLC accepts no liability for the content referenced in this activity.

*Collection and use of your personal information*

The submission of certain personally identifiable information is necessary to award and track the credits participants may earn after completing the program. Required items include first name, last name, degree, and email address. These items are necessary. Participants are also required to complete an evaluation of the CME activity. All evaluative information submitted is collected and retained, and used by Paradigm Medical Communications, LLC to continuously improve the learning experience.

Paradigm Medical Communications, LLC does not transfer, sell, or share personal information with outside parties. Paradigm Medical Communications, LLC will not otherwise disclose personal information unless required to in a legal process. Paradigm Medical Communications, LLC staff members review individual test results to the extent necessary to record and track participants’ credits.

**Contact Us**If you have any questions or comments, please email us at *contactus@paradigmmc.com*.

